# Multi-Modal PET and MR Imaging in the Hen’s Egg Test-Chorioallantoic Membrane (HET-CAM) Model for Initial In Vivo Testing of Target-Specific Radioligands

**DOI:** 10.3390/cancers12051248

**Published:** 2020-05-15

**Authors:** Gordon Winter, Andrea B. F. Koch, Jessica Löffler, Mika Lindén, Christoph Solbach, Alireza Abaei, Hao Li, Gerhard Glatting, Ambros J. Beer, Volker Rasche

**Affiliations:** 1 Department of Nuclear Medicine, Ulm University Medical Center, 89081 Ulm, Germany; andrea.koch@uni-ulm.de (A.B.F.K.); jessica.loeffler@uni-ulm.de (J.L.); christoph.solbach@uniklinik-ulm.de (C.S.); ambros.beer@uniklinik-ulm.de (A.J.B.); 2 Core Facility Small Animal Imaging, Ulm University Medical Center, 89081 Ulm, Germany; alireza.abaei@uni-ulm.de (A.A.); hao.li@uni-ulm.de (H.L.); 3 Department of Inorganic Chemistry II, Ulm University, 89081 Ulm, Germany; mika.linden@uni-ulm.de; 4 Department of Nuclear Medicine, Medical Radiation Physics, Ulm University Medical Center, 89081 Ulm, Germany; gerhard.glatting@uni-ulm.de; 5 Internal Medicine II, Ulm University Medical Center, 89081 Ulm, Germany

**Keywords:** PSMA, HET-CAM, chick embryo, PET, MRI, multi-modal imaging

## Abstract

The validation of novel target-specific radioligands requires animal experiments mostly using mice with xenografts. A pre-selection based on a simpler in vivo model would allow to reduce the number of animal experiments, in accordance with the 3Rs principles (reduction, replacement, refinement). In this respect, the chick embryo or hen’s egg test–chorioallantoic membrane (HET-CAM) model is of special interest, as it is not considered an animal until day 17. Thus, we evaluated the feasibility of quantitative analysis of target-specific radiotracer accumulation in xenografts using the HET-CAM model and combined positron emission tomography (PET) and magnetic resonance imaging (MRI). For proof-of-principle we used established prostate-specific membrane antigen (PSMA)-positive and PSMA-negative prostate cancer xenografts and the clinically widely used PSMA-specific PET-tracer [^68^Ga]Ga-PSMA-11. Tracer accumulation was quantified by PET and tumor volumes measured with MRI (*n* = 42). Moreover, gamma-counter analysis of radiotracer accumulation was done ex-vivo. A three- to five-fold higher ligand accumulation in the PSMA-positive tumors compared to the PSMA-negative tumors was demonstrated. This proof-of-principle study shows the general feasibility of the HET-CAM xenograft model for target-specific imaging with PET and MRI. The ultimate value for characterization of novel target-specific radioligands now has to be validated in comparison to mouse xenograft experiments.

## 1. Introduction

The development of target-specific radiolabeled probes like on the basis of small molecules, peptides, antibodies, or nanoparticles, always requires information on biodistribution and in particular on the level of specific binding of the novel probe to the target in the in vivo situation, which means specific radiotracer accumulation in the target-expressing region of interest [1,2,3,4,5]. Up to now, animal experiments are necessary to obtain this information mostly using mice with xenografts expressing the specific target and often also target-negative xenografts as controls. However, alternative methods to these animal experiments are of high interest regarding animal welfare according to the 3Rs principles (reduction, replacement, refinement), and also to potentially speed up the development of novel radioligands by simpler and nonetheless effective screening tools.

In this respect, the hen’s egg test-chorioallantoic membrane (HET-CAM) model is a promising alternative for animal experiments, as in many countries, chick embryos are not considered live animals before embryo development day (EDD) 17 or hatching. Thus, in contrast to animal studies, the HET-CAM model does not rise legal authorization issues if the embryos are sacrificed accordingly. Considering the fact that there is some dissent in the available literature concerning the point at which the chick embryo starts to feel pain, it is recommended to initiate the experiments as early as possible and to use methods with minimal burden [6,7,8,9,10,11,12,13,14]. The CAM is a well-suited platform for conducting experiments because of its textures and function as well as the complex character of its vascular system e.g., to study tumor growth and metastasis formations, tissue grafts, drug delivery, and toxicological analysis [15,16,17]. The CAM of fertilized chicken eggs is formed between EDD 4–10 of avian development. The mesodermal layers of the chorion and allantois fuse and build the CAM that generates a rich vascular network with a gas and waste exchange [18,19,20]. As a relatively simple, quick and inexpensive model, HET-CAM has already been used for studies on, e.g., angiogenesis [21], cancer progression [22], pharmacology [23], and radiotherapy [24]. It is also well suited for xenograft studies, as the chick embryo is not fully immunocompetent at early development stages. T-cells can be detected on embryo development day (EDD) 11 and B cells on EDD12 [25,26]. The immune system components further diversify until EDD18 when the embryo is fully immunocompetent [27].

Recently, several studies have been published about tumor imaging in the HET-CAM model, focusing on MRI compounds [28] or therapeutic effects after specific and non-specific accumulation [29,30], demonstrating the vast potential of these method. Our group has focused on optimization of conditions for in ovo imaging, e.g., cooling of the embryo and light fumigation with isoflurane to reduce motion artifacts [31,32,33]. There are also several publications on positron emission tomography (PET) imaging in chick embryos [23,34,35,36,37,38,39], but the models were neither combined with MRI nor were they extensively studied for quantitative tracer evaluation. To the best of our knowledge, the analysis of target-specific accumulation of radiotracers by combined PET and MR imaging in ovo has not yet been described before.

It was the objective of this proof-of-principle study to evaluate the feasibility of the combination of high-resolution MR imaging and highly sensitive PET imaging in ovo in a HET-CAM xenograft model system for evaluation of target-specific radiotracer accumulation. As target for our model system, we chose the prostate-specific membrane antigen (PSMA or glutamate carboxypeptidase II (EC 3.4.17.21)) [40] as it is often overexpressed in human prostate carcinoma (PCa) and thus of special clinical relevance). As specific radioligand we chose the clinically widely used PET tracer [^68^Ga]Ga-PSMA-11 [41,42,43]. The well-established PCa tumor cell line LNCaP [44] and its derivative LNCaP C4-2 [45] are known for high PSMA expression and served as PSMA-positive (PSMA^+^) xenografts [46,47,48,49,50]. As negative control for evaluation of target-specificity in the same embryo, we chose the also well-established PSMA-negative (PSMA^−^) PCa cell line PC-3 [51].

## 2. Results

### 2.1. Methodological Aspects

For all chick embryos (*n* = 66) MR scans were successfully performed with adequate quality for further analysis. Detailed information about presence or absence of tumors and thus successful tumor growth and the tumor volume could be provided.

**Figure 1:**
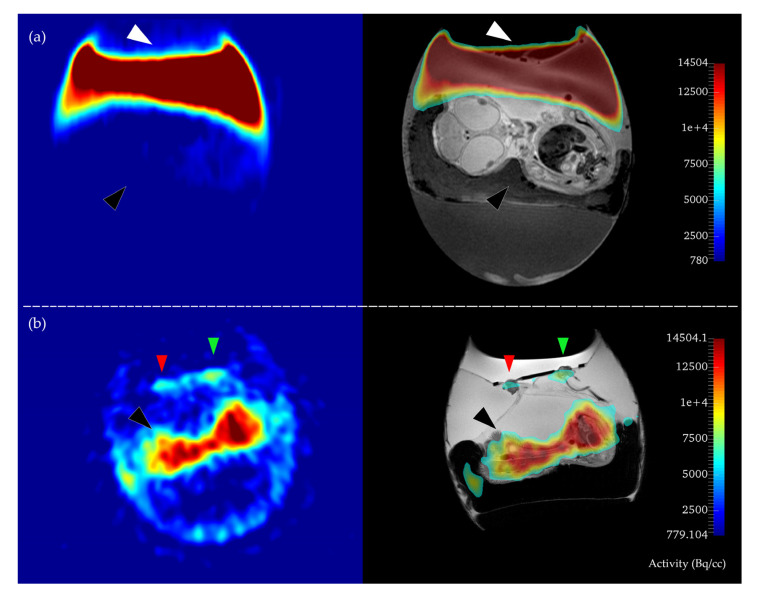
Examples of failed (**a**) and successful (**b**) systemic injection of the radiotracer. The positron emission tomography (PET) image (left side) and the respective PET/MR overlay (right side) are depicted. In (**a**), no tracer accumulation in the embryo can be observed (black arrow), while the whole tracer activity is pooled outside the system on top of the CAM, (white arrow). Thus, this embryo was excluded from further analysis. In (**b**) regular accumulation of the radiotracer in the body of the embryo can be seen (black arrow) without major paravasation. Note also the different intensities of radiotracer accumulation in the xenografts (green and red arrows = PSMA pos., PSMA neg).

Concerning PET, 42 chick embryos (64%) were available for final analysis and 24 chick embryos (36%) had to be excluded from further analysis because of the following reasons: In 14 (21%) cases the systemic intravascular injection of [^68^Ga]Ga-PSMA-11 was not successful and thus there was no detectable signal in the embryo in PET imaging (Figure 1). Four more chick embryos had to be excluded despite successful injection, because of extended paravasation of radiotracer adjacent to the tumor areas and consecutive spillover of activity which made a meaningful analysis of tracer accumulation in the tumors either by PET or gamma-counter measurements impossible. For two chick embryos (3%) no data were available because of technical problems. Four further chick embryos had to be excluded because of failure of tumor growth of the PSMA-positive cell line.

Minor paravasation was noted in 5 of the 42 analyzed chick embryos, however they were included for evaluation as ex vivo gamma counter measurements after rigorous cleansing of the xenografts were still possible despite a potential bias of the in vivo PET data due to spillover. In 5 of the 42 analyzed chick embryos, the PSMA-negative PC-3 cell line did not grow successfully and only the background activity could be analyzed (termed as “CAM”).

In summary, 42 chick embryos could be analyzed both by PET and MR: 37 chick embryos with both PSMA-positive and PSMA-negative xenografts (34 with LNCaP C4-2 + PC-3, 3 with LNCaP + PC-3) and 5 chick embryos with only the PSMA-positive xenograft (LNCaP C4-2 + CAM). The complete data are listed in detail in the Table S1.

### 2.2. In Ovo MR and PET Imaging

In MRI, the mean tumor volumes of PSMA-positive and PSMA-negative tumors were not significantly different (*p* = 0.06) with LNCaP C4-2 (32.4 ± 7.8 mm^3^), LNCaP (29.4 ± 0.1 mm^3^), and PC-3 (30.0 ± 2.3 mm^3^).

In the static PET measurements, the radiotracer accumulation in PSMA-positive tumors was significantly higher (mean 1.36 ± 0.68 MBq/cc; median 1.29 MBq/cc) than in the negative control tumors (mean 1.08 ± 0.61 MBq/cc, median = 0.99 MBq/cc; *p* = 0.04) and compared to the background (CAM; mean 0.88 ± 0.44 MBq/cc).

When analyzing the ratio of tracer accumulation in the PSMA-positive tumors versus PSMA-negative tumors, the ratio was above 1 in the majority of cases (23/37, 62%) with a mean ratio of (2.1 ± 1.4): [LNCaP C4-2 / PC-3] (2.1 ± 1.4, 1.9), [LNCaP / PC-3] (1.9 ± 0.8, 1.9) (mean ± SD, median).

**Figure 2:**
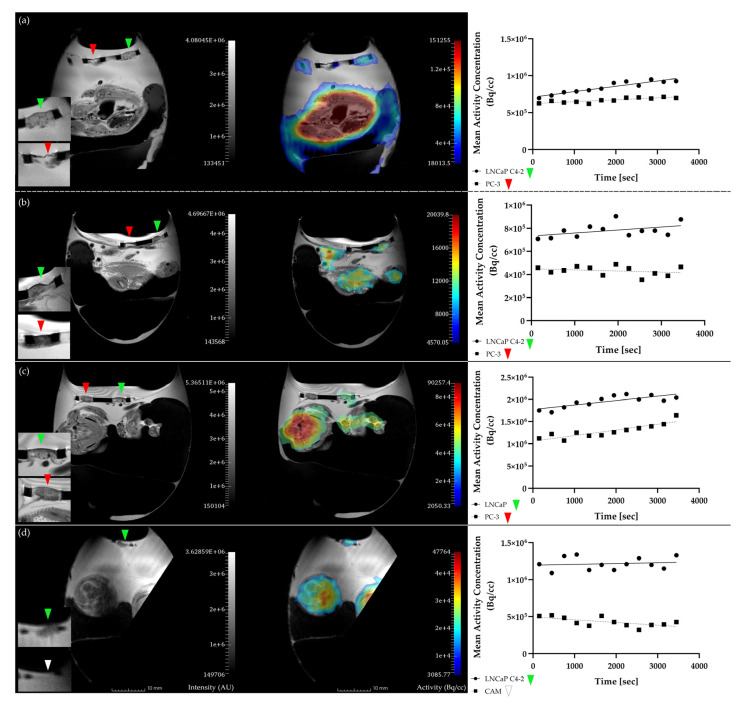
Magnetic resonance (MR) images (T2-weighted, TSE [28], left panel) and PET/MR fusion images (middle panel) of hen’s egg test–chorioallantoic membrane (HET-CAM) xenograft experiments with the corresponding time–activity curves for the respective PET region of interest (right panel). The PSMA-positive tumors can be clearly visualized in the MR image in all cases ((**a**,**b**,**d**): LNCaP C4-2, (**c**): LNCaP), whereas the negative tumor PC-3 can only be seen in examples (**a**–**c**) and did not grow in example d (PSMA^+^ → green arrow; PSMA^−^ → red arrow; CAM → white arrow). Note the more intense accumulation in the PSMA-positive tumors as compared to the PSMA-negative tumors, suggesting target-specific tracer accumulation.

We also performed dynamic measurements of radiotracer accumulation over time, however, as measurements normally started 10 min after injection, no perfusion peak can be observed in the time–activity curves and thus quantitative kinetic modelling was not performed (Figure 2). In a qualitative analysis of accumulation patterns, a constant activity level was observed for LNCaP C4-2, LNCaP, and PC-3 in 19, 2, and 22 curves, respectively. An increase of activity over time was observed in LNCaP C4-2, LNCaP, and PC-3 for 12, 1, and 9 chick embryos, while in 8, 0, and 6 embryos the activity decreased over time. The high variability of PET data corresponds with the qualitative evaluation of the time-activity-curves.

In the body of the embryo, the activity was heterogeneously distributed with highest accumulation in the heart region (see Figure 1 and Figure 2; [52]).

All data are listed in detail in Table S3.

### 2.3. Gamma Counter-Based Evaluation

**Figure 3:**
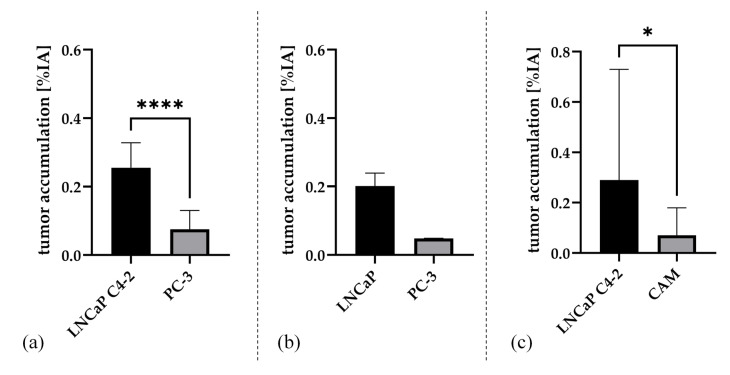
Mean and standard deviation of the three groups after paired evaluation of the tumor accumulation [%IA] test for (**a**) [LNCaP C4-2 + PC-3] (*n* = 34), (**b**) [LNCaP + PC-3] (*n* = 3), and (**c**) [LNCaP C4-2 + CAM] (*n* = 5). Significance of the pairwise comparison is indicated (*p*-values: **** *p* < 0.0001; * *p* < 0.05).

Absolute radiotracer accumulation evaluated ex vivo by gamma counter measurements showed a significantly higher accumulation in the PSMA-positive tumors in comparison to the PSMA-negative tumors (*n* = 37, (0.27 ± 0.16) %IA all PSMA^+^, (0.11 ± 0.11) %IA all PSMA^−^ PC-3; *p* < 0.0001). When separately analyzing the subgroup of LNCaP C4-2 (*n* = 32, mean (0.28 ± 0.17) %IA; median: 0.24 %IA) and excluding the five tumors with more intense spillover and thus potentially impaired data quality, the accumulation still was significantly higher compared to PC-3 (*p* < 0.001). Even in the five experiments with a more intense spillover, the activity ratios still were substantially higher in the PSMA^+^ tumors compared to the PSMA-negative tumors (3.7; 3.2; 1.5; 4.6; 4.2, mean ± SD = 3.4 ± 1.2, *p* = 0.01). Tracer accumulation was also substantially higher in the small subgroup of LNCaP versus PC-3 ((0.20 ± 0.03) %IA vs. (0.12 ± 0.11) %IA), however did not reach statistical significance because of the low number of experiments (*n* = 3, *p* = 0.25). Tracer accumulation was also significantly higher in PSMA-positive tumors compared to background (CAM only) mean 0.34 [LNCaP C4-2 + CAM] *n* = 5, *p* = 0.0313. Results of the subgroup evaluations are presented in Figure 3. Data for single experiments are listed in Table S2.

When using the MR-derived tumor volumes for calculation of the radioactivity concentration in %IA per gram, the values of PSMA-positive tumors were also significantly higher compared to PSMA-negative tumors (mean 9.3 %IA/g, mean 3.8 %IA/g, *p* < 0.0001). This resulted in high ratios of activity concentration of PSMA-positive versus negative tumors (mean ± SD) of 4.0 ± 3.9 [LNCaP C4-2 vs. PC-3], 4.9 ± 0.8 [LNCaP vs. PC-3], and also versus background with 24.8 ± 48.5 [LNCaP C4-2 vs. CAM]. Furthermore, the activity concentration ratios of the tumor activity in comparison to the total activity in the chick embryo (tumor/total) were determined (Table S4). Here also the PSMA-positive tumors (5.4 ± 3.2) showed a significantly higher ratio compared to the PSMA-negative tumors (mean 2.2 ± 2.1; *p* < 0.0001).

**Figure 4:**
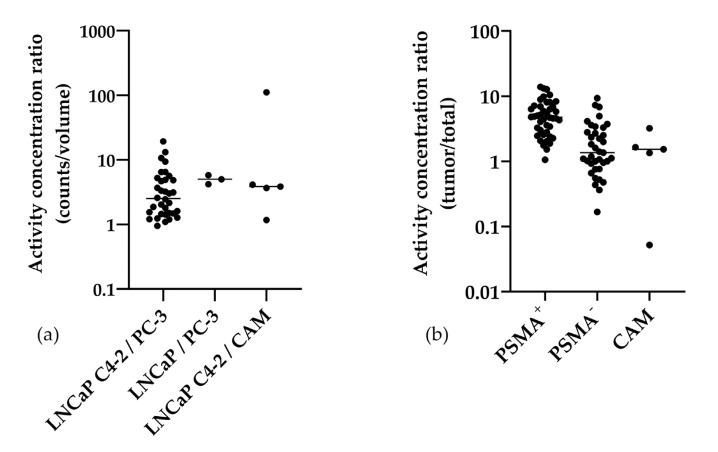
Scatter plots of the activity concentration ratios of the three experimental groups (PSMA^+^/PSMA^−^) in logarithmic scale (**a**) and the activity concentration ratios of the tumor area and the total chick embryo (**b**). Each experiment is depicted as a single dot, the median is shown. Notably, all ratios but one had a value greater than 1 for the direct comparison of the tumor entities (**a**). All values of PSMA^+^ in comparison to the total chick embryo were >1 (**b**).

The distribution of the data is visualized in a logarithmic scatter plot (Figure 4). The data are summarized in Table 1.

For the comparison of the results with the mouse xenograft model, several corresponding literature data were compiled in Table 2. Data on tumor accumulation of [^68^Ga]Ga-PSMA-11 and further PSMA-specific tracers were listed with regard to their accumulation in the tumor cell lines used in this study. In cases where the PSMA-positive and PSMA-negative tumor cell lines were available, the activity concentration ratio was calculated and additionally reported in the Table.

### 2.4. Immunohistochemistry for PSMA Detection

**Figure 5:**
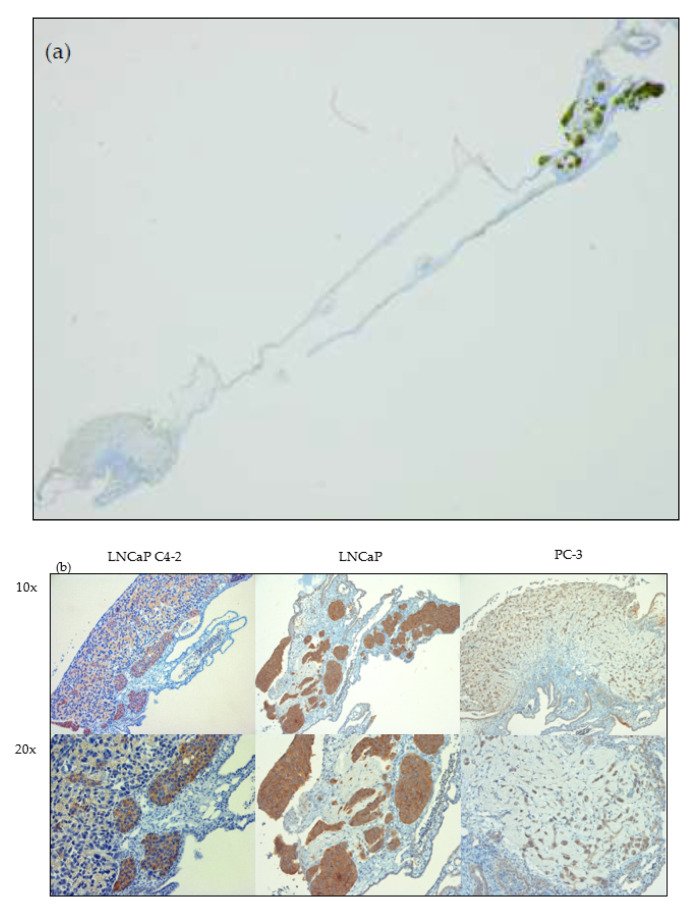
Immunohistochemical staining of HET-CAM tumor xenograft sections using an anti-PSMA antibody. Sections of each cell line specific tumor xenograft were used as an example. In image (**a**) an overview section is depicted. PC-3 and LNCaP tumor xenografts were stained simultaneously. In part (**b**) two magnifications (10×; 20×) of the tumor entities are depicted and an intense staining of the PSMA protein is clearly visible in the sections of LNCaP C4-2 and LNCaP (brown color), while a weak staining is visible for PC-3.

Immunohistochemical staining was performed to verify PSMA expression in the PSMA^+^ tumor entities in contrast to the PSMA^−^ control. In Figure 5 sections of the tumor cell lines are depicted in two magnifications as well as an overview demonstrating. In all sections a clear separation of the tumor tissue from the surrounding CAM is possible. An intense positive staining could be observed for the PSMA-positive tumors of LNCAP C4-2 and LNCAP. No substantial staining was observed for PSMA-negative PC-3 tumor cells with only faint staining of single areas, which is probably due to known PSMA expression in neoangiogenesis.

## 3. Discussion

In this feasibility study we demonstrated successful imaging and analysis of target-specific radiotracer accumulation in a HET-CAM xenograft model by combined PET and MRI, using the PSMA-specific PET tracer [^68^Ga]Ga-PSMA-11 and PSMA-positive and PSMA-negative tumor cell lines. Based on these promising data, further validation studies of the HET-CAM model compared to standard mice xenograft experiments are justified and warranted.

### 3.1. Analysis of In Ovo Target-Specific Binding of [^68^Ga]Ga-PSMA-11 and Comparison to Reported Data in Mice

We deliberately chose PSMA-expression as the model target structure for our proof-of-principle study as it is of paramount clinical relevance in PCA imaging and well established PSMA-positive and negative cell lines are available. As PET radiotracer we used the clinically widely used PSMA-specific probe [^68^Ga]Ga-PSMA-11. We could show significantly higher tracer accumulation in PSMA^+^ tumors compared to PSMA^−^ tumors by in vivo PET as well as in ex vivo gamma counter measurements, which clearly suggests PSMA-specific radiotracer accumulation. Moreover, a significantly higher ratio of accumulation in the tumor versus the embryo was demonstrated for the PSMA^+^ tumors compared to PSMA^−^ tumors, which is another indicator for PSMA-specific tracer accumulation. The results were comparable in androgen sensitive and androgen insensitive PSMA^+^ tumors, however the activity concentration ratio for LNCaP C4-2 versus PC-3 was slightly lower, possibly caused by a lower PSMA expression in LNCaP C4-2 compared to LNCaP which is reported in the literature [66,67,68].

We also successfully performed dynamic scanning in the HET-CAM model for analysis of time–activity curves (TACs), however no quantitative analysis was performed because of the missing perfusion phase as measurements could not be started earlier than 10 min. p.i., because the injection is too difficult to be performed in the scanner. Qualitative evaluation did not reveal substantial differences in the patterns of the TACs between PSMA^+^ and PSMA^−^ tumors. Although a direct comparison with the literature is difficult because of the later start of measurements, the principle feasibility of the technique could be shown.

In various publications, [^68^Ga]Ga-PSMA-11 and other PSMA-specific radioligands have been analyzed by in vivo studies in mice. In Table 2 animal experiments are listed using the LNCaP, LNCaP C4-2 or PC-3 cell line for comparison to our data. Comparing the absolute values and the ratios of tracer accumulation in PSMA^+^ and PSMA^−^ tumors of all ligands listed, the results of our HET-CAM experiments are very comparable and within the same order of magnitude, which further corroborates our data. It is of interest that similar to our results in the HET-CAM model, a minor ligand uptake was also observed for PC-3 tumors in mice. At first glance this is surprising as it was clearly demonstrated in various in vitro studies that the PC-3 cell line is PSMA-negative [69,70]. However, under in vivo conditions this might be explained by PSMA expression in neoangiogenesis [71,72,73,74]. Furthermore, Laidler et al. had demonstrated that PSMA expression can be reestablished in PC-3 by using the basic fibroblast growth factor (bFGF or FGF2) [75]. The bFGF plays a pivotal role in the developmental processes in the chick embryo and [76,77,78] and was specifically demonstrated in the chorioallantoic fluid [79]. Thus, a low expression of PSMA might have been induced in some PC-3 tumor cells by bFGF from the chick embryo. Taken together these factors might explain the minor accumulation of the ligand in PC-3 xenografts as well as the weak staining in our immunohistochemical assays. Further validation of this hypothesis by Western blotting will be relevant for future studies.

### 3.2. MR Imaging, Tumor Volumetry, and PET-MR Image Fusion

The high-resolution MR images and fusion with the PET data not only facilitated the anatomic localization of radiotracer accumulation, but also provided detailed information about successful tumor growth and tumor volume. This was of special relevance as in some cases, the cells did not adequately grow after inoculation and thus no macroscopic tumor was formed. This is a problem also encountered in mice xenografts. In the mouse model tumor growth could easily be checked by palpation, whereas it is more challenging in the HET-CAM model. Without the MRI information, PET might have misinterpreted a missing signal in an area with no tumor growth as lack of specific tracer accumulation in the “tumor” as it does not provide detailed anatomic information. However, with the combined information from MRI cases of inadequate tumor growth could be identified and either excluded from analysis or categorized as background (CAM only). In cases of adequate tumor development, MRI was able to provide precise data on the tumor localization, extent, and volume, which is crucial for an exact PET and gamma counter analysis. For PET measurements it is very helpful for correct placement of the respective VOI over the tumor area, especially in case of no or only little uptake, because e.g., if the VOI was drawn to big, the uptake of the tumor would have been underestimated. Moreover, the MRI derived volumes were used for the gamma counter-based calculations of the radiotracer concentration in the tumor tissue. In MRI no significant difference in the tumor volumes of PSMA^+^ and PSMA^−^ tumors were observed. This further corroborates our hypothesis that the differences in tracer uptake in PET in PSMA^+^ and PSMA^−^ tumors are mainly due to differences in target-specific binding and not just caused by differences in tumor size. This also explains the gamma counter analysis, in which the absolute values of the tracer uptake (%IA) corresponded well with the activity concentrations (%IA/gram).

With the capability of MRI to provide multiple image contrasts even in the small HET-CAM tumors [28], its potential to provide further important physiological and molecular information (e.g., perfusion, diffusion, and tissue relaxation properties) for a more detailed tumor characterization is of major interest for future studies. The required immobilization of the embryo has been proven possible by various means, with simple cooling of the embryo at 4 °C showing excellent results and no effect on the survival of the embryo [31,32]. Within the duration of the presented experiments, no effect on the growth of the tumor or the embryo was observed.

### 3.3. Methodological and Logistical Aspects of the HET-CAM Xenograft Model

Only 64% (*n* = 42) of the prepared chick embryos could be used for the final evaluation. The major factor for exclusion was failed systemic injection of the radiotracer in 14 cases as the correct injection of the substance into the small CAM vessels is challenging. Bleeding after injection is another major challenge in this model as radiotracer in the blood may be distributed across the CAM adjacent to the tumor areas and thus impairing the measurements. Bleeding may be caused by choosing comparatively large vessels for injection in order to be able to work with the smallest standard cannulas (30G). Even if an injection was well administered, the wound in the blood vessel was relatively large. The use of cotton buds and liquid dressing spray stopped bleeding in most cases. However, moving the embryo could cause the wound to open again. Moreover, in chick embryos between EDD12-EDD16, wound healing processes and vasoconstriction are still not fully developed [80,81,82]. Smaller blood vessels could be used for the injection, but this increased the likelihood of failed injections. In five experiments a minor spillover of blood after injection was observed and the embryos were not excluded from analysis. While the activity ratio (PSMA^+^/PSMA^−^) based on PET imaging for these chick embryos was <1, the corresponding gamma counter data provided values >1. It can be assumed that by washing of the excised tumors the remaining residual activity could be successfully removed from the surface, while the PET data were biased by spill-in of unspecific activity.

In summary, the dropout rate was higher as compared to our experience with mice xenografts. However, we also noted a substantial learning curve and with increasing expertise we expect a lower failure rate in future studies. Despite these methodological challenges, the HET-CAM model still appears very attractive from the cost and administrative burden point of view. Costs for purchasing and keeping the model are only a fraction of the costs of similar studies in mice. Since no approval for animal testing is required in most countries, a study based on HET-CAM can start much faster than a comparable study in mice. The HET-CAM model allows the easy introduction of various tumor models, not only based on human cell lines but also on rodent cell lines or patient-derived material [15,29,30,31,39,83,84,85]. This facilitates the transfer from an established mouse model to the HET-CAM model for at least initial evaluation. However, further studies on optimization and validation against standard tumor models are certainly required before more widespread use.

### 3.4. Limitations

A limitation of the model is the comparably short time available for performing experiments, since chick embryos hatch on day 21. Considering that embryos start developing a sensation of pain not in the first trimester but most likely before EDD15 and experiments should be stopped latest at EDD18 [6,7,8,9,10,11,12,13,14], the time window for the measurements is reduced to about 4–7 days. An earlier start of the measurements is conceivable, provided that the tumors have reached a reasonable size.

A limitation of our study might be that the tumor weight was estimated based on the MR-measured tumor volume and not directly weighed as after excision of the tumor, the measured weight was unreliable because of the small size and wetness of the washed tumor. However, ex vivo measured absolute activities and activity concentrations correlated well with each other and also with the in vivo PET data thus suggesting that this was of minor relevance.

Another potential limitation is the lack of blocking experiments to further validate the specificity of tracer uptake in the PSMA^+^ tumors. Unfortunately, blocking experiments could not yet be done, as double injection in the HET-CAM model is quite challenging and single injection with samples separated by air bubbles might kill the embryo. However, we believe that the use of target-positive and negative tumors also allows a good characterization of the specificity of tracer uptake and is also commonly used in mouse xenograft experiments [61,86,87]. Moreover, the tracer [^68^Ga]Ga-PSMA-11 is very well characterized and our data are in line with reported data in the same tumor cell lines in mice.

Moreover, the exact quantification of tracer accumulation in small structures based on PET data is challenging because of the comparatively low resolution of PET systems and the influence of the partial-volume effect (PVE). The PVE, as described by Soret et al. [88], depends on the finite spatial resolution (affected by detector construction, image reconstruction) and image sampling (voxel size). For small lesions, signal is spread out leading to an underestimation of the activity concentration and an overestimation of tumor size, but also activity from the outside, e.g., from nearby blood vessels or the embryo, spills in and possibly biases the results. Tumor entities with a size less than three times the full width at half maximum (FWHM) are affected by PVE. The diameter of the tumor was limited by the 5 mm silicone rings and it usually grew up to 1.5–2.5 mm in depth. The optimal spatial resolution of the Focus 120 PET scanner is 1.13 mm FWHM (tangential, FBP) in the center field of view [89]. Tumor size of at least 3.39 mm and smaller would therefore be affected by PVE. Consequently, the PET-based activity concentration was underestimated and for a PV correction the system-specific properties of the respective PET system must therefore be considered in future HET-CAM studies. In our study PSMA^+^ and PSMA^−^ tumor xenografts were of similar volume. Even though the absolute uptake is underestimated, analysis of the uptake differences still allows correct interpretation of the data. A PV correction might be considered in future studies after determination of the exact correction factors for tumors of different sizes and intensities of uptake in relation to the surrounding tissue, which is challenging. Moreover, a PV correction also has disadvantages as it also always increases the noise in the data. Therefore, the effort and benefit of a corresponding complex calculation must be carefully considered. An attractive strategy would be the autoradiographic imaging of the tumors after extraction because the higher resolution of this technique leads to a considerably decreased PVE. In case no additional gamma counter measurement will be performed, then a very efficient PV correction must be implemented.

Finally concerning the LNCaP tumors only five embryos were evaluated. Initially an equal number of androgen sensitive and insensitive PSMA^+^ tumors should be examined. However, after we experienced a relatively high dropout rate in the first experiments, we primarily used one PSMA^+^ cell line as the major focus was on demonstrating differences in PSMA^+^ and PSMA^−^ tumors. Still we wanted to show the data of the other cell line to the readers as well despite the lower number of experiments.

## 4. Materials and Methods

### 4.1. Cell Culture

The androgen-dependent prostate carcinoma (PCa) cell line LNCaP [44] and its androgen-independent derivative LNCaP C4-2 [45] as well as PC-3 [51] are used as model system for HET-CAM xenograft design. While the cell lines LNCaP and LNCaP C4-2 are known for the high expression level of the PSMA protein [46], there is no detectable PSMA expression in the PC-3 cell line, which therefore was used as negative control. The human PCa cell lines LNCaP (ACC256, DSMZ, Braunschweig, Germany), LNCaP C4-2 (ViroMed Laboratories, Minnetonka, USA), and PC-3 (ACC465, DSMZ, Braunschweig, Germany) were cultivated as described elsewhere [90]. For HET-CAM xenograft experiments, cells were counted using a Neubauer hemocytometer and the respective number of cells was applied onto the CAM in silicone rings as described in the HET-CAM-model section.

### 4.2. HET-CAM Model

**Figure 6:**
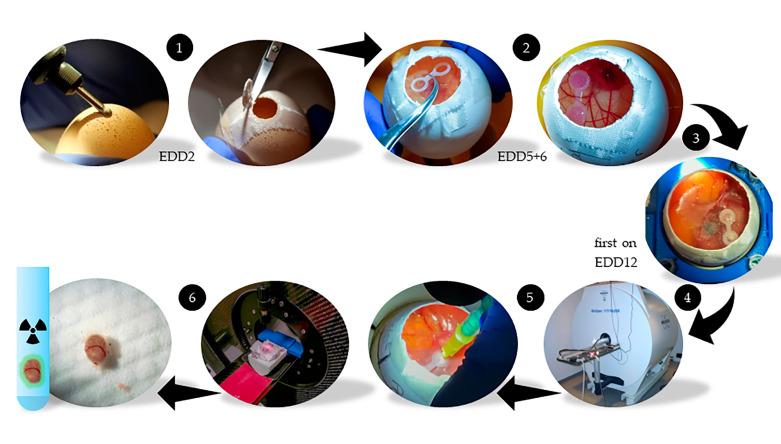
Workflow of the HET-CAM xenograft model. On the arrival day (EDD0) the eggs were incubated. On EDD2 the eggs were carefully opened (**1**). On days EDD5 and EDD6 the silicone rings were positioned, and the tumor cells were applied (**2**). The development of the tumors and the chick embryos was regularly checked (**3**). Between EDD12 and EDD16 three to five measurements per day were performed. Experiments started with high-resolution MRI (**4**). Immediately afterwards the ligand was systemically administered into a blood vessel of the CAM and the corresponding PET scan was performed (**5**). After PET the tumors were carefully excised, washed and the accumulated radioactivity was detected in a gamma counter (**6**). The embryos would not recover from the tumor excision and were sacrificed immediately.

The method we used was similar to already published HET-CAM models [17,83,91,92,93,94]. Fertilized chicken eggs (LSL Rhein-Main Geflügelvermehrungsbetriebe GmbH & Co.KG, Dieburg, Germany) were defined upon start of incubation on embryo development day 0 (EDD0). The eggshell was carefully opened on EDD2 using a cordless drill and scissors (Figure 6). The small opening was closed with clinical tape (Leukosilk, BSNmedical, Hamburg, Germany) allowing continuous gas and humidity exchange. After five days of incubation at 37.8 °C and 65% of relative humidity (EDD5), silicone double-rings were placed on the membrane of the opened chicken eggs. Xenografts were established by pipetting the PCa cell lines LNCaP C4-2 (1 × 10^6^ cells; PSMA^+^), LNCaP (1 × 10^6^ cells; PSMA^+^), and PC-3 (1 × 10^6^ cells; PSMA^−^) with Matrigel (40%, *v*/*v*) in an overall volume of 30 µL into the silicone rings on EDD6. Two groups of cell line combinations in the HET-CAM model were prepared: LNCaP C4-2 + PC-3 (61 chick embryos) and LNCaP + PC-3 (5 chick embryos). When only LNCaP C4-2 and no PC-3 tumor was established the embryo were used as a third group [LNCaP C4-2 + CAM], validating the CAM in the PC-3 area of the silicone rings. Tumor growth and embryo health were daily monitored by visual inspection. The MR and PET imaging experiments were performed between EDD12 and EDD16. For MRI scans the chick embryos were cooled for 70 to 120 min at 4 °C prior to the measurement start to minimize movement artefacts, according to the protocols of Bain et al. and Zuo et al. [31,32]. The eggshell opening was widened to the maximum without affecting the membrane and 1 mL of 0.9% NaCl solution was pipetted to shift the interface between the membrane surface and the atmosphere away from the tumor. During image acquisition the chick embryo was fumigated with 3% isoflurane [33]. For PET imaging the NaCl solution was completely removed and the surface carefully dried. A 30G needle (B. Braun, Melsungen, Germany) was bend and combined with a 1-mL syringe for the injection of 75 µL of [^68^Ga]Ga-PSMA-11 (1.3 µg/mL) into a chorioallantoic membrane blood vessel. On average an activity of 1.7 ± 1.5 MBq, in median 1.1 MBq was injected. To stop the bleeding after injection a cotton bud was held on the injection side and liquid bandage spray (Opsite, Smith & Nephew GmbH, Hamburg, Germany) was pipetted on the dried surface. The complete chick embryo and afterwards also needle, syringe, and cotton buds were measured in an activity meter (CRC-12, Capintec, NJ, USA) to determine the successfully applied radioactivity which was set to 100% injected activity [%IA] for quantification calculation. Events, where blood was slightly distributed across the membrane or near/into the tumor areas were protocolled and considered during the evaluation of the data, while large spillover lead to exclusion from evaluation in four cases. The embryos have been sacrificed after tumor excision.

### 4.3. MR and PET Imaging

The precursor PSMA-HBED-CC (PSMA-11) was purchased from ABX GmbH (Radeberg, Germany). The radiopharmaceutical [^68^Ga]Ga-PSMA-HBED-CC ([^68^Ga]Ga-PSMA-11) was produced as recently published [41,42]. For radiolabeling, a 50 mCi (1850 MBq) ^68^Ge/^68^Ga radionuclide generator was used (iThemba LABS, South Africa).

The eggs were placed in a custom-build holder for enabling PET-MRI registration by specific fiducials filled with 50 µl CuSO_4_ and 1 µl of [^68^Ga]Ga-PSMA-11.

The implemented protocols for MR measurements were based on the publications of Zuo et al. [28,31]. Imaging was performed on an 11.7 Tesla small animal MRI system (Bruker BioSpec 117/16, Bruker Biospin, Ettlingen, Germany). Data were obtained with a 72 mm quadrature volume T/R resonator.

Tumor volume, location, and structure were assessed by applying a high-resolution T2-weighted multislice rapid acquisition with relaxation enhancement (RARE) sequence. Scan parameters were as follows: TR/TE = 4320/45 ms, matrix size = 650 × 650, in-plane resolution = 77 × 91 μm^2^, slice thickness = 0.5 mm, no interslice gap, RARE factor = 8 and NSA = 4. The number of slices was adapted to the tumor size and ranged from 15 to 30 slices. The acquisition time for a single high-resolution RARE scan was between 15 and 35 min depending on the number of slices required to cover a sufficient volume of interest, covering both, the tumors and the chick embryos. 

For PET imaging a dynamic 60 min scan was performed on a small animal PET scanner (Focus120, Siemens Medical Solutions, Inc., Erlangen, Germany). The Focus120 has a high spatial resolution (<1.3 mm) and high sensitivity (about 7%) with a bore size (12 cm diameter and 7.6 cm axial length) [89]. These list-mode files were processed to obtain histogrammed data (sinograms) for a single 60 min image and an additional image of 12 dynamic frames of 5 min each. The reconstruction algorithm OSEM3D/MAP using 4 OSEM2D, 2 OSEM3D, and 18 MAP iterations was implemented. Partial volume correction was not performed to avoid additional noise in the data.

MRI and PET data were fused by fiducial registration using the 3Dslicer software (ver. 4.10.2) [95] and the software Amide (ver. 1.0.4). Additional data conversion was achieved using the Vinci software. (ver. 5.02) [96]. The area of the silicone rings and the tumor entities were selected in the MR images using the segmentation tool to have a visual support for activity detection in the specific tumor regions in PET images. For time-activity evaluation of the PET scans elliptical cylinder VOIs were drawn in the areas of the inner silicone rings with a size of 5 × 5 × D mm^3^, where *D* was at least 1.5 mm and adapted to the respective tumor size. VOI analysis provided voxel-based mean data for each 5 min time frame.

### 4.4. Gamma Counter Quantification and Evaluation

After washing (1 min), the tumors were analyzed with a gamma-counter COBRA II (Perkin Elmer) to accurately quantify the accumulated radioactivity. Quantification of the radioactivity in the tumor was based on these gamma-counter measurements followed by decay correction and calculation of the fraction in comparison to the applied activity (percent injected activity, %IA). The quotient of the activity ratios and the tumor volume ratios of the paired PSMA-positive and PSMA-negative xenografts [radioactivity in the tumor (PSMA^+^/PSMA^−^) / tumor volume (PSMA^+^/PSMA^−^)] was calculated to provide the value for evaluation of the ligand accumulation. Values > 1 indicate a higher accumulation in the PSMA-positive tumor regions. Using data from the gamma counter and volume determination based on MR images, the accumulated activity in %IA/g was calculated for comparison with literature data. The calculation is based on the definition of 1000 mm^3^ = 1 g, whereby the density of water as the main component in the cells is approximated.

Wilcoxon matched-pairs signed rank tests were performed using GraphPad Prism (ver. 8.4.2 for Windows, GraphPad Software, San Diego, California USA, www.graphpad.com). A *p*-value < 0.05 was assumed as statistically significant.

### 4.5. Immunohistochemistry

Tumor excision tissues were fixated in 4% formaldehyde in phosphate-buffered saline (PBS) and paraffin-embedded. Sections were deparaffinized using xylene (3×; 5 min) and rehydrated by washing in descending alcohol solutions. To reveal the masked epitopes antigen retrieval was obtained by boiling citrate-based antigen unmasking solution (1:100; 5 min; pH 6.0; Vector Laboratories, CA). The cooled sections were washed for 1 min using 30% H_2_O_2_ in PBS solution to quench endogenous peroxidase activity followed by an additional washing in PBS for 5 min. To prevent background-staining phosphate-buffered albumin (PBA; 1% BSA in PBS) was applied for 60 min followed by treatment using 2% normal serum (blocking serum) (Vector Laboratories, CA) in PBA for 30 min at room temperature. The mouse monoclonal primary antibody YPSMA-1 (ab19071, Abcam, Cambridge, UK) was applied in a dilution of 1:800 in PBA and the sections were incubated overnight at 4 °C in a wet chamber. After washing in PBS (2×, 5 min), biotinylated secondary antibody (Vector Laboratories, CA) diluted 1:200 in PBA was applied for 60 min in a wet chamber at room temperature. Sections were rinsed in PBS (2×, 5 min) and incubated with avidin/biotin-based VECTASTAIN^®^ Elite^®^ ABC Kit (Peroxidase (HRP) working solution (Vector Laboratories, CA) for 30 min at room temperature. Following an additional washing in PBS (2×, 5 min), Vector^®^ DAB peroxidase substrate (Vector Laboratories, CA) was applied for 10 min at room temperature. The substrate was removed by washing with water (2×, 5 min) and counterstained using hematoxylin solution modified according to Gill III (Merck KGaA, Darmstadt, Germany). After dehydration by ascending alcohol steps (2 min) and toluene (2×, 2 min), slides were mounted (Fisher Chemical™ Permount™; Thermo Fisher Scientific, Germany).

## 5. Conclusions

Our feasibility study suggests that the HET-CAM xenograft model using PET and MR imaging is promising for the initial assessment of specific radiotracer accumulation under in vivo conditions and the results of our study are in line with the reported data in mice xenografts. The model has many advantages compared to the mouse model, e.g., lower costs, less administrative work, a short experimental start-up time, and less maintenance effort. On the other hand, its application is limited by the rather short time window for the experimental procedures, and technical challenges like injecting into the rather small CAM vessels. However, the time window is only of minor importance in the context of the intended approach for the initial analysis of new radioligands and the technical challenges can be overcome with appropriate experience. Now further comparative studies for validation of our data have to be performed. If successfully established, combined PET-MR imaging using the HET-CAM xenograft model would allow for a substantial reduction of animal experiments in line with the 3Rs principles.

## Figures and Tables

**Table 1:** Medians of the different calculated ratios and the respective *p*-values. *p*-values < 0.05 are assumed statistically significant. Low sample numbers were not sufficient for statistical evaluation (ns = not significant), but the results still provide information about the tendency in the experimental groups. LNCaP C4-2 + PC-3 (*n* = 34); LNCaP + PC-3 (*n* = 3); LNCaP C4-2 + CAM (*n* = 5).

Tumor Entities	Activity Ratio (PSMA^+^/PSMA^−^) [Median]	Activity Concentration Ratio (PSMA^+^/PSMA^−^) [Median]	Activity Concentration Ratio (Tumor/Total) [Median]	*p* Value (Two Tailed)
LNCaP C4-2	3.0	2.5	4.8	<0.0001 (****)
PC-3			1.5	
LNCaP	5.0	5.0	4.6	0.250 (ns)
PC-3			0.9	
LNCaP C4-2	4.1	3.9	5.8	0.0625 (ns)
CAM			1.5	

**Table 2:** Animal experiments in mice using [^68^Ga]Ga-PSMA-11 (Glu-NH-CO-NH-Lys(Ahx)-HBED-CC) or a similar PSMA-specific tracer in comparison to the present HET-CAM study (mean ± SD). The activity concentration ratios have been calculated based on the published data.

Ligand	Tumor Accumulation [%IA/g]			Activity Concentration Ratio (PSMA^+^/PSMA^−^)	Incubation Time [h]	Ref.
	LNCaP	LNCaP C4-2	PC-3			
^68^Ga-PSMA-11	-	9.27 ± 5.61	4.02 ± 3.78	2.3; (4.0) ^†^	1	HET-CAM ^§^
^68^Ga-PSMA-11	6.95 ± 1.31	-	1.45 ± 0.41	4.8; (4.9) ^†^	1	HET-CAM ^§^
^68^Ga-PSMA-11	7.70 ± 1.45	-	1.30 ± 0.12	5.9	1	[41]
^68^Ga-PSMA-11	4.89 ± 1.34	-	1.30 ± 0.12	3.8	1	[42]
^68^Ga-PSMA-11	7.28 ± 0.82	-	1.21 ± 0.07	6.0	1	[53]
^68^Ga-PSMA-11	5.89 ± 2.82	-	-	-	1	[54]
^68^Ga-PSMA-11	12.75 ± 2.49	-	-	-	1	[55]
^68^Ga-PSMA-11	8.91 ± 0.86	-	-	-	1	[56]
^68^Ga-PSMA-11	8.42 ± 1.40	-	-	-	1	[57]
^68^Ga-PSMA-11	7.19 ± 0.86	-	-	-	1	[57]
^68^Ga-PSMA-11	8.20 ± 2.44	-	-	-	1	[57]
^68^Ga-PSMA-11	8.67 ± 1.97	-	-	-	1	[57]
^68^Ga-PSMA-11-Dimer	8.22 ± 1.78	-	0.93 ± 0.53	8.8	1	[42]
^18^F-PSMA-11	55.7 ± 11.8	-	3.1 ± 0.9	18.0	6	[58]
^18^F-PSMA-1007	8.04 ± 2.39	-	1.05 ± 0.11	7.7	1	[59]
[^11^C]DCMC	8.73 ± 0.73	-	1.65 ± 0.29	5.3	0.5	[60]
[^11^C]DCIT	5.07 ± 1.02	-	1.78 ± 0.63	2.8	0.5	[60]
^68^Ga-HE_0_	2.38 ± 0.05	-	0.84 ± 0.18	2.8	1	[54]
^68^Ga-HE_1_	2.41 ± 1.24	-	2.67 ± 1.25	0.9	1	[54]
^68^Ga-HE_2_	10.66 ± 4.19	-	1.99 ± 0.40	2.5	1	[54]
^68^Ga-HE_3_	3.22 ± 0.22	-	1.66 ± 0.41	1.9	1	[54]
^203^Pb-PSMA-CA012	-	8.4 ± 3.7	-	-	1	[61]
DUPA-^99m^Tc	11.2	-	-	-	4	[62]
^68^Ga-CHX-A″-DTPA conjugate	~3 *	-	-	-	1	[63]
^68^Ga-CHX-A″-DOTA conjugate	~7 *	-	-	-	1	[63]
^68^Ga-PSMA-617	8.47 ± 4.09	-	-	-	1	[64]
^177^Lu-PSMA-617	11.20 ± 4.17	-	-	-	1	[64]
^64^Cu-PSMA-617	-	-	3.47 ± 0.48	-	24	[65]

† ratios of the mean; (mean of the ratios); § present study; * values estimated from diagram.

## Supplementary Materials

The following are available online at https://www.mdpi.com/2072-6694/12/5/1248/s1, Table S1: Overview on chick embryo, Table S2: Gamma counter raw data and activity concentration ratios (PSMA^+^/PSMA^−^), Table S3: Gamma counter data [%IA and activity ratios (PSMA^+^/PSMA^−^) based on %IA and on PET data, Table S4: Values of the activity concentration ratios (Tumor/total) and the uptake concentration in [%IA/g].

## References

[1] De Silva R.A., Kumar D., Lisok A., Chatterjee S., Wharram B., Venkateswara Rao K., Mease R., Dannals R.F., Pomper M.G., Nimmagadda S. (2018). Peptide-based ^68^Ga-PET radiotracer for imaging PD-L1 expression in cancer. Mol. Pharm..

[2] Amor-Coarasa A., Kelly J., Ponnala S., Vedvyas Y., Nikolopoulou A., Williams C., Jin M.M., David Warren J., Babich J.W. (2018). [^18^F]RPS-544: A PET tracer for imaging the chemokine receptor CXCR4. Nucl. Med. Biol..

[3] Lindner T., Loktev A., Altmann A., Giesel F., Kratochwil C., Debus J., Jager D., Mier W., Haberkorn U. (2018). Development of quinoline-based theranostic ligands for the targeting of fibroblast activation protein. J. Nucl. Med..

[4] Beckford Vera D.R., Smith C.C., Bixby L.M., Glatt D.M., Dunn S.S., Saito R., Kim W.Y., Serody J.S., Vincent B.G., Parrott M.C. (2018). Immuno-PET imaging of tumor-infiltrating lymphocytes using zirconium-89 radiolabeled anti-CD3 antibody in immune-competent mice bearing syngeneic tumors. PLoS ONE.

[5] Stockhofe K., Postema J.M., Schieferstein H., Ross T.L. (2014). Radiolabeling of nanoparticles and polymers for PET imaging. Pharmaceuticals.

[6] Leary S., Underwood W., Anthony R., Cartner S., Grandin T., Greenacre C., Gwaltney-Brant S., McCrackin M.A., Meyer R., Miller D. (2020). AVMA Guidelines for the Euthanasia of Animal: 2020 Edition.

[7] Deutscher Bundestag (2017). Zum Schmerzempfinden von Hühnerembryonen. Deutscher Bundestag/Wissenschaftliche Dienste 2017, Umwelt, Naturschutz, Reaktorsicherheit, Bildung und Forschung.

[8] Aleksandrowicz E., Herr I. (2015). Ethical euthanasia and short-term anesthesia of the chick embryo. ALTEX.

[9] Bjørnstad S., Austdal L.P.E., Roald B., Glover J.C., Paulsen R.E. (2015). Cracking the Egg: Potential of the Developing Chicken as a Model System for Nonclinical Safety Studies of Pharmaceuticals. J. Pharmacol. Exp. Ther..

[10] Campbell M.L.H., Mellor D.J., Sandoe P. (2014). How should the welfare of fetal and neurologically immature postnatal animals be protected?. Anim. Welfare.

[11] Eide A.L., Glover J.C. (1995). Development of the longitudinal projection patterns of lumbar primary sensory afferents in the chicken embryo. J. Comp. Neurol..

[12] Eide A.L., Glover J.C. (1997). Developmental dynamics of functionally specific primary sensory afferent projections in the chicken embryo. Anat. Embryol. (Berl.).

[13] Rosenbruch M. (1994). Early stages of the incubated chicken egg as a model in experimental biology and medicine. ALTEX Altern. Anim. Exp..

[14] Rosenbruch M. (1997). The sensitivity of chicken embryos in incubated eggs. ALTEX Altern. Anim. Exp..

[15] Harris J.J. (1958). The human tumor grown in the egg. Ann. N. Y. Acad. Sci..

[16] Ebert J.D. (1954). The Effects of Chorioallantoic Transplants of Adult Chicken Tissues on Homologous Tissues of the Host Chick Embryo. Proc. Natl. Acad. Sci. USA.

[17] Ribatti D. (2016). The chick embryo chorioallantoic membrane (CAM). A multifaceted experimental model. Mech. Dev..

[18] Rahn H., Paganelli C.V., Ar A. (1974). The avian egg: Air-cell gas tension, metabolism and incubation time. Respir. Physiol..

[19] Romanoff A.L. (1960). The Extraembryonic Membranes. The Avian Embryo. Structural and Functional Development.

[20] Schlatter P., Konig M.F., Karlsson L.M., Burri P.H. (1997). Quantitative study of intussusceptive capillary growth in the chorioallantoic membrane (CAM) of the chicken embryo. Microvasc. Res..

[21] Mangieri D., Nico B., Benagiano V., De Giorgis M., Vacca A., Ribatti D. (2008). Angiogenic activity of multiple myeloma endothelial cells in vivo in the chick embryo chorioallantoic membrane assay is associated to a down-regulation in the expression of endogenous endostatin. J. Cell Mol. Med..

[22] Lokman N.A., Elder A.S., Ricciardelli C., Oehler M.K. (2012). Chick chorioallantoic membrane (CAM) assay as an in vivo model to study the effect of newly identified molecules on ovarian cancer invasion and metastasis. Int. J. Mol. Sci..

[23] Haller S., Ametamey S.M., Schibli R., Müller C. (2015). Investigation of the chick embryo as a potential alternative to the mouse for evaluation of radiopharmaceuticals. Nucl. Med. Biol..

[24] Dünker N., Jendrossek V. (2019). Implementation of the Chick Chorioallantoic Membrane (CAM) Model in Radiation Biology and Experimental Radiation Oncology Research. Cancers.

[25] Janse E.M., Jeurissen S.H. (1991). Ontogeny and function of two non-lymphoid cell populations in the chicken embryo. Immunobiology.

[26] Vargas A., Zeisser-Labouebe M., Lange N., Gurny R., Delie F. (2007). The chick embryo and its chorioallantoic membrane (CAM) for the in vivo evaluation of drug delivery systems. Adv. Drug Deliv. Rev..

[27] Moreno-Jiménez I., Hulsart-Billstrom G., Lanham S.A., Janeczek A.A., Kontouli N., Kanczler J.M., Evans N.D., Oreffo R.O. (2016). The chorioallantoic membrane (CAM) assay for the study of human bone regeneration: A refinement animal model for tissue engineering. Sci. Rep..

[28] Zuo Z., Syrovets T., Wu Y., Hafner S., Vernikouskaya I., Liu W., Ma G., Weil T., Simmet T., Rasche V. (2017). The CAM cancer xenograft as a model for initial evaluation of MR labelled compounds. Sci. Rep..

[29] Vu B.T., Shahin S.A., Croissant J., Fatieiev Y., Matsumoto K., Le-Hoang Doan T., Yik T., Simargi S., Conteras A., Ratliff L. (2018). Chick chorioallantoic membrane assay as an in vivo model to study the effect of nanoparticle-based anticancer drugs in ovarian cancer. Sci. Rep..

[30] Wittig R., Rosenholm J.M., Haartman E.v., Hemming J., Genze F., Bergman L., Simmet T., Lindén M., Sahlgren C. (2014). Active targeting of mesoporous silica drug carriers enhances γ-secretase inhibitor efficacy in an in vivo model for breast cancer. Nanomedicine.

[31] Zuo Z., Syrovets T., Genze F., Abaei A., Ma G., Simmet T., Rasche V. (2015). High-resolution MRI analysis of breast cancer xenograft on the chick chorioallantoic membrane. NMR Biomed..

[32] Bain M.M., Fagan A.J., Mullin J.M., McNaught I., McLean J., Condon B. (2007). Noninvasive monitoring of chick development in ovo using a 7T MRI system from day 12 of incubation through to hatching. J. Magn. Reson. Imaging.

[33] Heidrich A., Würbach L., Opfermann T., Saluz H.P. (2011). Motion-artifact-free in vivo imaging utilizing narcotized avian embryos in ovo. Mol. Imaging Biol..

[34] Dupertuis Y.M., Delie F., Cohen M., Pichard C. (2015). In ovo method for evaluating the effect of nutritional therapies on tumor development, growth and vascularization. Clin. Nutr. Exp..

[35] Würbach L., Heidrich A., Opfermann T., Gebhardt P., Saluz H.P. (2012). Insights into bone metabolism of avian embryos in ovo via 3D and 4D ^18^F-fluoride positron emission tomography. Mol. Imaging Biol..

[36] Warnock G., Turtoi A., Blomme A., Bretin F., Bahri M.A., Lemaire C., Libert L.C., Seret A.E., Luxen A., Castronovo V. (2013). In vivo PET/CT in a human glioblastoma chicken chorioallantoic membrane model: A new tool for oncology and radiotracer development. J. Nucl. Med..

[37] Steinemann G., Dittmer A., Schmidt J., Josuttis D., Fahling M., Biersack B., Beindorff N., Jolante Koziolek E., Schobert R., Brenner W. (2019). Antitumor and antiangiogenic activity of the novel chimeric inhibitor animacroxam in testicular germ cell cancer. Mol. Oncol..

[38] Freesmeyer M., Kuehnel C., Opfermann T., Niksch T., Wiegand S., Stolz R., Huonker R., Witte O.W., Winkens T. (2018). The use of ostrich eggs for in ovo research: Making preclinical imaging research affordable and available. J. Nucl. Med..

[39] Zlatopolskiy B.D., Zischler J., Schäfer D., Urusova E.A., Guliyev M., Bannykh O., Endepols H., Neumaier B. (2018). Discovery of 7-[^18^F]fluorotryptophan as a novel positron emission tomography (PET) probe for the visualization of tryptophan metabolism in vivo. J. Med. Chem..

[40] Davis M.I., Bennett M.J., Thomas L.M., Bjorkman P.J. (2005). Crystal structure of prostate-specific membrane antigen, a tumor marker and peptidase. Proc. Natl. Acad. Sci. USA.

[41] Eder M., Schäfer M., Bauder-Wüst U., Hull W.E., Wangler C., Mier W., Haberkorn U., Eisenhut M. (2012). ^68^Ga-complex lipophilicity and the targeting property of a urea-based PSMA inhibitor for PET imaging. Bioconjug. Chem..

[42] Schäfer M., Bauder-Wüst U., Leotta K., Zoller F., Mier W., Haberkorn U., Eisenhut M., Eder M. (2012). A dimerized urea-based inhibitor of the prostate-specific membrane antigen for ^68^Ga-PET imaging of prostate cancer. EJNMMI Res..

[43] Miksch J., Bottke D., Krohn T., Thamm R., Bartkowiak D., Solbach C., Bolenz C., Beer M., Wiegel T., Beer A.J. (2020). Interobserver variability, detection rate, and lesion patterns of ^68^Ga-PSMA-11-PET/CT in early-stage biochemical recurrence of prostate cancer after radical prostatectomy. Eur. J. Nucl. Med. Mol. Imaging.

[44] Horoszewicz J.S., Leong S.S., Kawinski E., Karr J.P., Rosenthal H., Chu T.M., Mirand E.A., Murphy G.P. (1983). LNCaP model of human prostatic carcinoma. Cancer Res..

[45] Wu H.C., Hsieh J.T., Gleave M.E., Brown N.M., Pathak S., Chung L.W. (1994). Derivation of androgen-independent human LNCaP prostatic cancer cell sublines: Role of bone stromal cells. Int. J. Cancer.

[46] Taylor R.M., Severns V., Brown D.C., Bisoffi M., Sillerud L.O. (2012). Prostate cancer targeting motifs: Expression of α_v_β_3_, neurotensin receptor 1, prostate specific membrane antigen, and prostate stem cell antigen in human prostate cancer cell lines and xenografts. Prostate.

[47] Troyer J.K., Beckett M.L., Wright G.L. (1995). Detection and characterization of the prostate-specific membrane antigen (PSMA) in tissue extracts and body fluids. Int. J. Cancer.

[48] Denmeade S.R., Sokoll L.J., Dalrymple S., Rosen D.M., Gady A.M., Bruzek D., Ricklis R.M., Isaacs J.T. (2003). Dissociation between androgen responsiveness for malignant growth vs. Expression of prostate specific differentiation markers PSA, hK2, and PSMA in human prostate cancer models. Prostate.

[49] Smith-Jones P.M., Vallabahajosula S., Goldsmith S.J., Navarro V., Hunter C.J., Bastidas D., Bander N.H. (2000). In vitro characterization of radiolabeled monoclonal antibodies specific for the extracellular domain of prostate-specific membrane antigen. Cancer Res..

[50] Schulke N., Varlamova O.A., Donovan G.P., Ma D., Gardner J.P., Morrissey D.M., Arrigale R.R., Zhan C., Chodera A.J., Surowitz K.G. (2003). The homodimer of prostate-specific membrane antigen is a functional target for cancer therapy. Proc. Natl. Acad. Sci. USA.

[51] Kaighn M.E., Narayan K.S., Ohnuki Y., Lechner J.F., Jones L.W. (1979). Establishment and characterization of a human prostatic carcinoma cell line (PC-3). Investig. Urol..

[52] Winter G. (2020). In PET Images a Major Accumulation of [^68^Ga]Ga-PSMA-11 Was Observed in the Developing Heart and Liver Region of the Chick Embryo. A Detailed Analysis Will Be in the Focus of Future Studies.

[53] Wang Y., Shao G., Wu J., Cui C., Zang S., Qiu F., Jia R., Wang Z., Wang F. (2018). Preparation of ^68^Ga-PSMA-11 with a synthesis module for micro PET-CT imaging of PSMA expression during prostate cancer progression. Contrast Media Mol. Imaging.

[54] Liolios C., Schäfer M., Haberkorn U., Eder M., Kopka K. (2016). Novel bispecific PSMA/GRPr targeting radioligands with optimized pharmacokinetics for improved PET imaging of prostate cancer. Bioconjug. Chem..

[55] Greifenstein L., Engelbogen N., Lahnif H., Sinnes J.-P., Bergmann R., Bachmann M., Rösch F. (2020). Synthesis, labeling and preclinical evaluation of a squaric acid containing PSMA inhibitor labeled with ^68^Ga: A comparison with PSMA-11 and PSMA-617. ChemMedChem.

[56] Kuo H.T., Pan J., Zhang Z., Lau J., Merkens H., Zhang C., Colpo N., Lin K.S., Benard F. (2018). Effects of linker modification on tumor-to-kidney contrast of ^68^Ga-labeled PSMA-targeted imaging probes. Mol. Pharm..

[57] Rousseau E., Lau J., Kuo H.-T., Zhang Z., Merkens H., Hundal-Jabal N., Colpo N., Lin K.-S., Bénard F. (2018). Monosodium glutamate reduces ^68^Ga-PSMA-11 uptake in salivary glands and kidneys in a preclinical prostate cancer model. J. Nucl. Med. Off. Publ. Soc. Nucl. Med..

[58] Boschi S., Lee J.T., Beykan S., Slavik R., Wei L., Spick C., Eberlein U., Buck A.K., Lodi F., Cicoria G. (2016). Synthesis and preclinical evaluation of an Al^18^F radiofluorinated Glu-urea-Lys(Ahx)-HBED-CC PSMA ligand. Eur. J. Nucl. Med. Mol. Imaging.

[59] Cardinale J., Schafer M., Benesova M., Bauder-Wust U., Leotta K., Eder M., Neels O.C., Haberkorn U., Giesel F.L., Kopka K. (2017). Preclinical evaluation of ^18^F-PSMA-1007, a new prostate-specific membrane antigen ligand for prostate cancer imaging. J. Nucl. Med..

[60] Foss C.A., Mease R.C., Fan H., Wang Y., Ravert H.T., Dannals R.F., Olszewski R.T., Heston W.D., Kozikowski A.P., Pomper M.G. (2005). Radiolabeled small-molecule ligands for prostate-specific membrane antigen: In vivo imaging in experimental models of prostate cancer. Clin. Cancer Res..

[61] Dos Santos J.C., Schäfer M., Bauder-Wüst U., Lehnert W., Leotta K., Morgenstern A., Kopka K., Haberkorn U., Mier W., Kratochwil C. (2019). Development and dosimetry of ^203^Pb/^212^Pb-labelled PSMA ligands: Bringing “the lead” into PSMA-targeted alpha therapy?. Eur. J. Nucl. Med. Mol. Imaging.

[62] Kularatne S.A., Wang K., Santhapuram H.K., Low P.S. (2009). Prostate-specific membrane antigen targeted imaging and therapy of prostate cancer using a PSMA inhibitor as a homing ligand. Mol. Pharm..

[63] Wüstemann T., Bauder-Wüst U., Schäfer M., Eder M., Benesova M., Leotta K., Kratochwil C., Haberkorn U., Kopka K., Mier W. (2016). Design of internalizing PSMA-specific Glu-ureido-based radiotherapeuticals. Theranostics.

[64] Benesova M., Schäfer M., Bauder-Wüst U., Afshar-Oromieh A., Kratochwil C., Mier W., Haberkorn U., Kopka K., Eder M. (2015). Preclinical evaluation of a tailor-made DOTA-conjugated PSMA inhibitor with optimized linker moiety for imaging and endoradiotherapy of prostate cancer. J. Nucl. Med..

[65] Han X.D., Liu C., Liu F., Xie Q.H., Liu T.L., Guo X.Y., Xu X.X., Yang X., Zhu H., Yang Z. (2017). ^64^Cu-PSMA-617: A novel PSMA-targeted radio-tracer for PET imaging in gastric adenocarcinoma xenografted mice model. Oncotarget.

[66] Fan X., Wang L., Guo Y., Tu Z., Li L., Tong H., Xu Y., Li R., Fang K. (2015). Ultrasonic nanobubbles carrying anti-PSMA nanobody: Construction and application in prostate cancer-targeted imaging. PLoS ONE.

[67] Wang X., Ma D., Olson W.C., Heston W.D. (2011). In vitro and in vivo responses of advanced prostate tumors to PSMA ADC, an auristatin-conjugated antibody to prostate-specific membrane antigen. Mol. Cancer Ther..

[68] Michalska M., Schultze-Seemann S., Bogatyreva L., Hauschke D., Wetterauer U., Wolf P. (2016). In vitro and in vivo effects of a recombinant anti-PSMA immunotoxin in combination with docetaxel against prostate cancer. Oncotarget.

[69] Ghosh A., Wang X., Klein E., Heston W.D. (2005). Novel role of prostate-specific membrane antigen in suppressing prostate cancer invasiveness. Cancer Res..

[70] Yao V., Berkman C.E., Choi J.K., O’Keefe D.S., Bacich D.J. (2010). Expression of prostate-specific membrane antigen (PSMA), increases cell folate uptake and proliferation and suggests a novel role for PSMA in the uptake of the non-polyglutamated folate, folic acid. Prostate.

[71] Chang S.S., Reuter V.E., Heston W.D., Gaudin P.B. (2001). Metastatic renal cell carcinoma neovasculature expresses prostate-specific membrane antigen. Urology.

[72] Rhee H., Ng K.L., Tse B.W.-C., Yeh M.-C., Russell P.J., Nelson C., Thomas P., Samaratunga H., Vela I., Gobe G. (2016). Using prostate specific membrane antigen (PSMA) expression in clear cell renal cell carcinoma for imaging advanced disease. Pathology.

[73] Baccala A., Sercia L., Li J., Heston W., Zhou M. (2007). Expression of prostate-specific membrane antigen in tumor-associated neovasculature of renal neoplasms. Urology.

[74] Wernicke A.G., Kim S., Liu H., Bander N.H., Pirog E.C. (2017). Prostate-specific membrane antigen (PSMA) expression in the neovasculature of gynecologic malignancies: Implications for PSMA-targeted therapy. Appl. Immunohistochem. Mol. Morphol..

[75] Laidler P., Dulińska J., Lekka M., Lekki J. (2005). Expression of prostate specific membrane antigen in androgen-independent prostate cancer cell line PC-3. Arch. Biochem. Biophys..

[76] Joseph-Silverstein J., Consigli S.A., Lyser K.M., Ver Pault C. (1989). Basic fibroblast growth factor in the chick embryo: Immunolocalization to striated muscle cells and their precursors. J. Cell Biol..

[77] Danesi R., Del Bianchi S., Soldani P., Campagni A., La Rocca R.V., Myers C.E., Paparelli A., Del Tacca M. (1993). Suramin inhibits bFGF-induced endothelial cell proliferation and angiogenesis in the chick chorioallantoic membrane. Br. J. Cancer.

[78] Funakoshi Y., Matsuda S., Uryu K., Fujita H., Okumura N., Sakanaka M. (1993). An immunohistochemical study of basic fibroblast growth factor in the developing chick. Anat. Embryol. (Berl.).

[79] Flamme I., Schulze-Osthoff K., Jacob H.J. (1991). Mitogenic activity of chicken chorioallantoic fluid is temporally correlated to vascular growth in the chorioallantoic membrane and related to fibroblast growth factors. Development.

[80] Weiss P., Matoltsy A.G. (1957). Absence of wound healing in young chick embryos. Nature.

[81] Weiss P., Matoltsy A.G. (1959). Wound healing in chick embryos in vivo and in vitro. Dev. Biol..

[82] van Golde J.M., Mulder T.A., Scheve E., Prinzen F.W., Blanco C.E. (1999). Hyperoxia and local organ blood flow in the developing chick embryo. J. Physiol..

[83] DeBord L.C., Pathak R.R., Villaneuva M., Liu H.-C., Harrington D.A., Yu W., Lewis M.T., Sikora A.G. (2018). The chick chorioallantoic membrane (CAM) as a versatile patient-derived xenograft (PDX) platform for precision medicine and preclinical research. Am. J. Cancer Res..

[84] Armstrong P.B., Quigley J.P., Sidebottom E. (1982). Transepithelial invasion and intramesenchymal infiltration of the chick embryo chorioallantois by tumor cell lines. Cancer Res..

[85] Klingenberg M., Becker J., Eberth S., Kube D., Wilting J. (2014). The chick chorioallantoic membrane as an in vivo xenograft model for Burkitt lymphoma. BMC Cancer.

[86] Chen Y., Foss C.A., Byun Y., Nimmagadda S., Pullambhatla M., Fox J.J., Castanares M., Lupold S.E., Babich J.W., Mease R.C. (2008). Radiohalogenated prostate-specific membrane antigen (PSMA)-based ureas as imaging agents for prostate cancer. J. Med. Chem..

[87] Chen Y., Pullambhatla M., Foss C.A., Byun Y., Nimmagadda S., Senthamizhchelvan S., Sgouros G., Mease R.C., Pomper M.G. (2011). 2-(3-{1-carboxy-5-[(6-[^18^F]fluoro-pyridine-3-carbonyl)-amino]-pentyl}-ureido)-pen tanedioic acid, [^18^F]DCFPyL, a PSMA-based PET imaging agent for prostate cancer. Clin. Cancer Res..

[88] Soret M., Bacharach S.L., Buvat I. (2007). Partial-volume effect in PET tumor imaging. J. Nucl. Med..

[89] Kim J.S., Lee J.S., Im K.C., Kim S.J., Kim S.-Y., Lee D.S., Moon D.H. (2007). Performance measurement of the microPET Focus 120 scanner. J. Nucl. Med..

[90] Kramer L., Winter G., Baur B., Kuntz A.J., Kull T., Solbach C., Beer A.J., Linden M. (2017). Quantitative and correlative biodistribution analysis of ^89^Zr-labeled mesoporous silica nanoparticles intravenously injected into tumor-bearing mice. Nanoscale.

[91] Sharrow A.C., Ishihara M., Hu J., Kim I.H., Wu L. (2020). Using the chicken chorioallantoic membrane in vivo model to study gynecological and urological cancers. JoVE.

[92] Jefferies B., Lenze F., Sathe A., Truong N., Anton M., von Eisenhart-Rothe R., Nawroth R., Mayer-Kuckuk P. (2017). Non-invasive imaging of engineered human tumors in the living chicken embryo. Sci. Rep..

[93] Kunzi-Rapp K., Genze F., Kufer R., Reich E., Hautmann R.E., Gschwend J.E. (2001). Chorioallantoic membrane assay: Vascularized 3-dimensional cell culture system for human prostate cancer cells as an animal substitute model. J. Urol..

[94] Ribatti D. (2014). The chick embryo chorioallantoic membrane as a model for tumor biology. Exp. Cell Res..

[95] Fedorov A., Beichel R., Kalpathy-Cramer J., Finet J., Fillion-Robin J.C., Pujol S., Bauer C., Jennings D., Fennessy F., Sonka M. (2012). 3D slicer as an image computing platform for the quantitative imaging network. Magn. Reson. Imaging.

[96] Vollmar S., Hampl J.A., Kracht L., Herholz K. (2007). Integration of Functional Data (PET) into Brain Surgery Planning and Neuronavigation.

