# Supplementary material for: Multi-Modal PET and MR Imaging in the Hen’s Egg Test-Chorioallantoic Membrane (HET-CAM) Model for Initial In Vivo Testing of Target-Specific Radioligands
Source: Cancers (Basel). 2020 May 15;12(5):1248. doi: 10.3390/cancers12051248 (PMC7281765; doi:10.3390/cancers12051248)
Supplement: Supplementary file 1 [file cancers-12-01248-s001.pdf]

Supplementary Materials: Multi-Modal PET and MR Imaging in the Hen's Egg Test-Chorioallantoic Membrane (HET-CAM) Model for Initial *in Vivo* Testing of Target-Specific Radioligands

Gordon Winter, Andrea B. F. Koch, Jessica Löffler, Mika Lindén, Christoph Solbach, Alireza Abaei, Hao Li, Gerhard Glatting, Ambros J. Beer and Volker Rasche

Table S1. Overview on chick embryo selection.

Chick embryo Nr.	Tumor growth			Injection failed [F]	Technical problems [T]	Spillover
	LNCaP C4-2	PC-3	LNCaP			Protocolled [P] Excluded [X]
1	+	+				
2	+	+				
3	+	+				
4	+	-				
5	+	-				
6	+	+				X
7	+	+				X
8	-	-				
9	+	+		F		
10	+	+				
11	+	+				
12	+	+				
13	+	-				
14	-	-				
15	+	+				
16	+	-				
17	+	+				
18	+	-				
19	+	+		F		
20	+	-		F		
21	+	+				
22	+	+				
23	+	+				P
24	+	+				P
25	+	+		F		
26	+	+				
27	+	+				
28	+	+				
29	+	+				
30	-	+				
31	+	+				
32	+	+		F		
33	+	+			T	
34	+	+				X
35	+	+		F		
36	+	+				
37	-	-				
38	+	+				
39	+	+		F		
40	+	+				
41	+	+				
42	+	+				
43	+	+				
44	+	+				
45	+	+				P
46	+	+				X
47	+	+				
48	+	+		F		
49	+	+		F		
50	+	+				
51	+	+				
52	+	+				
53	+	+		F		

54	+	+				P
55	+	+			T	
56		+	+			P
57		+	+	F		
58		+	+			
59		+	+	F		
60		+	+			
61	+	+		F		
62	+	+				
63	+	+				
64	+	+		F		
65	+	+				
66	+	+				

Table S2. Gamma counter raw data and activity concentration ratios (PSMA+/PSMA-).

Chick embryo Nr.	Gamma Counter Raw Data [CPM]			Activity concentration ratio (PSMA$^+$/PSMA$^-$)		
	PSMA$^+$ (LNCaP C4-2 & LNCaP)	PC-3	Background (Mean)	LNCaP C4-2 + PC-3	LNCaP + PC-3	LNCaP C4-2 + CAM
1	19983.6	8155.4	40.0	2.45		
2	15653.8	2318.1	32.0	4.91		
3	109736.8	50694.6	85.0	2.16		
4	56910.3	11630.6	35.0			3.86
5	51309.2	13988.6	30.0			3.67
6	16516.8	13354.4	22.7			
7	10908.3	5160.7	26.0			
8	32069.8	31222.9	25.7			
9	1507.4	543.4	23.0			
10	14661.3	8156.5	21.2	1.80		
11	18547.1	11565.3	20.5	1.61		
12	9654.8	4752.5	28.0	2.04		
13	16550.2	4018.6	27.0			4.14
14	1526.7	5363.3	24.0			
15	15157.8	15854.8	29.0	0.95		
16	6672.8	5674.2	20.0			1.17
17	28799.6	24018	29.1	1.20		
18	15960.6	182	39.3			111.63
19	1224.6	308	28.3			
20	135	118	23.7			
21	12991.7	1859.2	30.7	5.66		
22	30678.2	10075.2	36.6	2.58		
23	28284.3	7633	29.0	3.69		
24	29730.6	9248.9	28.3	3.22		
25	8750.4	5588.6	27.3			
26	16359	3403	28.7	4.85		
27	13942.1	4041.2	25.5	3.03		
28	18633.3	12828.3	29.0	1.45		
29	42113.5	2193.6	28.5	19.36		
30	23011.6	571	20.8			
31	5795.5	3724.1	24.0	1.24		
32	221	211.4	25.8			
33	16401	1146.4	23.9			
34	2947.2	1483	24.8			
35	12513.1	4630.9	22.0			
36	13767.7	2633.2	23.6	5.25		
37	986.4	1990.4	26.0			
38	15174.8	13765.3	25.3	1.10		
39	18469.7	8422.5	26.0			
40	7336	5174.5	21.3	1.43		
41	2847.5	1851.7	23.0	1.55		
42	5306.4	4382.9	26.8	1.21		
43	46066.4	30551.9	28.3	1.51		
44	23202.5	7387.5	26.3	3.13		
45	45659.7	30685.3	27.7	1.49		
46	10293	3143.6	22.0			
47	5216.7	821.4	24.7	6.52		
48	1885.2	5351.1	26.0			

49	3431.5	3293.6	24.8				
50	5668.7	820.9	22.0		9.41		
51	8938.4	696.4	20.8		13.18		
52	173394	16234.8	24.5		10.70		
53	6304	3947.5	25.3				
54	17837.3	3824	22.3		4.70		
55	2817.2	750.9	19.6				
56	48464.4	11470.4	23.5			4.21	
57	2335.7	1321.9	22.5				
58	27994.9	5633.2	22.5			5.00	
59	30413.6	22952	24.0				
60	27239.8	4743.1	26.0			5.79	
61							
62	15706.7	5547.9	25.5		1.87		
63	60224.9	47028.4	26.2		1.27		
64	15326.9	312	26.1				
65	4549.8	1385.8	24.3		3.33		
66	2733.2	442	23.7		6.50		
				n	34	3	5
				**Mean**	4.01	5.00	24.89
				**SD**	3.98	0.79	48.50
				**Median**	2.52	5.00	3.86

Table S3. Gamma counter data [%IA/ and activity ratios (PSMA$^+$/PSMA$^-$) based on %IA and on PET data.

Chick embryo Nr.	Gamma Counter Data [%ID] [LNCaP C4-2+PC-3]		[LNCaP+PC-3]		[LNCaP C4-2+CAM]		Activity ratio (based on %ID) (PSMA$^+$/PSMA$^-$)			Activity ratio based on PET data (PSMA$^+$/PSMA$^-$)		
	LNCaP C4-2	PC-3	LNCaP	PC-3	LNCaP C4-2	CAM	LNCaP C4-2 + PC-3	LNCaP + PC-3	LNCaP C4-2 + CAM	LNCaP C4-2 + PC-3	LNCaP + PC-3	LNCaP C4-2 + CAM
1	0.13	0.05					2.6			2.4		
2	0.49	0.07					7.0			1.9		
3	0.28	0.13					2.2			1.8		
4					0.34	0.07			4.9			2.0
5					0.24	0.07			3.4			3.7
6												
7												
8												
9												
10	0.26	0.14					1.9			3.2		
11	0.11	0.07					1.6			1.5		
12	0.12	0.06					2.0			**0.6**		
13					0.73	0.18			4.1			2.5
14												
15	0.35	0.37					**0.9**			**1.0**		
16					0.09	0.08			1.1			**0.9**
17	0.43	0.37					1.2			**0.9**		
18					0.29	0.003			115.2			2.4
19												
20												
21	0.17	0.02					8.5			1.3		
22	0.25	0.08					3.1			1.3		
23	0.11	0.03					3.7			**0.3**		
24	0.32	0.10					3.2			1.2		
25												
26	0.11	0.02					5.5			2.3		
27	0.21	0.06					3.5			1.8		
28	0.07	0.05					1.4			2.1		
29	0.56	0.03					18.7			1.9		
30												
31	0.28	0.18					1.5			2.0		
32												
33												
34												
35												
36	0.44	0.08					5.2			2.9		
37												
38	0.19	0.17					1.1			2.3		
39												
40	0.14	0.10					1.4			**0.9**		
41	0.18	0.12					1.5			**0.4**		
42	0.33	0.27					1.2			**0.9**		
43	0.73	0.49					1.5			1.6		
44	0.62	0.20					3.1			**0.7**		
45	0.22	0.15					1.5			**0.3**		

46												
47	0.14	0.02					6.4			**0.5**		
48												
49												
50	0.42	0.06					7.0			7.9		
51	0.48	0.04					13.0			2.0		
52	0.42	0.04					10.6			1.1		
53												
54	0.28	0.06					4.6			**0.6**		
55												
56			0.20	0.05				4.2			**0.4**	
57												
58			0.24	0.05				4.9			1.3	
59												
60			0.17	0.03				5.7			2.5	
61												
62	0.30	0.11					2.8			**1.0**		
63	0.21	0.17					1.3			1.2		
64												
65	0.17	0.05					3.3			**1.0**		
66	0.05	0.01					6.3			1.1		
n	34	34	3	3	5	5	34	3	5	34	3	5
**Mean**	**0.28**	**0.12**	**0.20**	**0.04**	**0.34**	**0.08**	4.12	4.93	25.73	1.58	1.40	2.30
**SD**	**0.17**	**0.11**	**0.03**	**0.01**	**0.24**	**0.06**	3.88	0.75	50.03	1.34	1.05	1.01
**Median**	**0.26**	**0.08**	**0.20**	**0.05**	**0.29**	**0.07**	**2.96**	**4.92**	**4.06**	1.25	1.30	2.40

Table S4. Values of the Activity concentration ratios (Tumor/total) and the uptake concentration in [%IA/g].

Chick embryo Nr.	Total Activity in chick embryo [MBq]	LNCaP C4-2+PC-3				LNCaP+PC-3				LNCaP C4-2+CAM				Activity concentration ratio (Tumor/total)					
														LNCaP C4-2+PC-3		LNCaP+PC-3		LNCaP C4-2+CAM	
		LNCaP C4-2 [kBq/mm^3]	LNCaP C4-2 [%IA/g]	PC-3 [kBq/mm^3]	PC-3 [%IA/g]	LNCaP [kBq/mm^3]	LNCaP [%IA/g]	PC-3 [kBq/mm^3]	PC-3 [%IA/g]	LNCaP C4-2 [kBq/mm^3]	LNCaP C4-2 [%IA/g]	CAM [kBq/mm^3]	CAM [%IA/g]	LNCaP C4-2/total	PC-3/total	LNCaP/total	PC-3/total	LNCaP C4-2/total	CAM/total
1	2.27	0.10	4.30	0.04	1.78									2.46	1.02				
2	0.51	0.06	12.04	0.01	2.45									6.93	1.41				
3	5.92	0.56	9.39	0.26	4.40									5.38	2.52				
4	4.42									0.40	9.03	0.10	2.36					6.28	1.64
5	2.74									0.22	8.06	0.06	2.22					4.88	1.35
6																			
7																			
8																			
9																			
10	1.99	0.17	8.70	0.10	4.89									4.80	2.70				
11	2.51	0.09	3.74	0.06	2.35									1.89	1.19				
12	2.10	0.08	3.94	0.04	1.96									2.03	1.01				
13	0.40									0.10	24.94	0.02	6.07					13.20	3.21
14																			
15	0.60	0.07	11.74	0.07	12.49									6.86	7.30				
16	1.13									0.04	3.16	0.03	2.76					1.76	1.53
17	1.44	0.21	14.72	0.18	12.47									8.07	6.83				
18	0.78									0.08	9.68	0.00	0.09					5.80	0.05
19																			
20																			
21	1.06	0.04	3.43	0.01	0.60									2.07	0.36				
22	1.00	0.12	12.38	0.05	4.85									7.14	2.80				
23	3.67	0.14	3.86	0.04	1.06									2.40	0.66				
24	2.31	0.25	10.85	0.08	3.41									6.33	1.99				
25																			
26	1.87	0.07	3.79	0.01	0.80									2.26	0.47				
27	1.01	0.07	7.01	0.02	2.34									4.06	1.36				
28	3.41	0.08	2.29	0.05	1.60									1.53	1.07				
29	1.64	0.31	18.97	0.02	1.00									10.48	0.55				
30																			
31	0.37	0.03	7.50	0.02	6.14									4.40	3.60				
32																			
33																			
34														0.00	0.00				

35																			
36	0.49	0.07	14.90	0.01	2.87									8.34	1.61				
37																			
38	1.02	0.07	6.38	0.06	5.89									3.58	3.31				
39																			
40	0.82	0.04	4.77	0.03	3.38									2.57	1.83				
41	0.28	0.02	6.08	0.01	3.97									3.42	2.24				
42	0.30	0.03	11.13	0.03	9.34									5.88	4.93				
43	1.10	0.27	24.76	0.18	16.62									13.90	9.33				
44	1.18	0.25	20.97	0.08	6.78									12.74	4.12				
45	1.46	0.11	7.49	0.07	5.10									5.02	3.42				
46																			
47	0.72	0.03	4.66	0.01	0.73									2.79	0.44				
48																			
49																			
50	0.29	0.04	14.25	0.00	1.53									8.90	0.96				
51	0.79	0.13	16.19	0.01	1.24									9.84	0.76				
52	5.75	0.82	14.29	0.08	1.34									8.12	0.76				
53																			
54	0.85	0.08	9.48	0.02	2.04									5.19	1.12				
55																			
56	4.46					0.32	7.10	0.08	1.71							4.57	1.10		
57																			
58	1.59					0.13	8.18	0.03	1.65							4.56	0.92		
59																			
60	2.21					0.12	5.58	0.02	0.98							3.00	0.53		
61																			
62	0.83	0.06	6.67	0.03	3.61									4.31	2.33				
63	3.61	0.26	7.11	0.20	5.65									4.69	3.73				
64																			
65	0.33	0.02	5.68	0.01	1.73									3.28	1.00				
66	0.98	0.02	1.67	0.00	0.26									1.06	0.17				
**n**	42.00	34	**34**	34	**34**	3	**3**	3	**3**	5	**5**	5	**5**	34	34	3	3	5	5
**Mean**	1.72	0.14	**9.27**	0.06	**4.02**	0.19	**6.95**	0.04	**1.45**	0.17	**10.98**	0.04	**2.70**	4.68	2.02	4.05	0.85	6.38	1.56
**SD**	1.45	0.16	**5.61**	0.06	**3.78**	0.11	**1.31**	0.03	**0.41**	0.15	**8.22**	0.04	**2.15**	3.51	2.14	0.90	0.29	4.20	1.12
**Median**	1.12	0.08	**7.50**	0.03	**2.66**	0.13	**7.10**	0.03	**1.65**	0.10	**9.03**	0.03	**2.36**	**4.31**	**1.19**	**4.56**	**0.92**	**5.80**	**1.53**

© 2020 by the authors. Submitted for possible open access publication under the terms and conditions of the Creative Commons Attribution (CC BY) license (http://creativecommons.org/licenses/by/4.0/).
